# Editorial: New paradigm of attention and attention training: Mechanisms and applications

**DOI:** 10.3389/fnhum.2023.1122941

**Published:** 2023-01-20

**Authors:** Fushun Wang, Roy F. Baumeister, Yi-Yuan Tang

**Affiliations:** ^1^Institute of Brain and Psychological Sciences, Sichuan Normal University, Chengdu, China; ^2^School of Psychology, The University of Queensland, Brisbane, QLD, Australia; ^3^College of Health Solutions, Arizona State University, Tempe, AZ, United States

**Keywords:** attention, effort, consciousness, autonomic control, mental disorders, emotion, mindfulness

In recent years, the attentional research paradigms have been broadened from traditional laboratory studies to include rigorous theoretical explorations of attention and clinical application such as meditation. For example, attention was thought to include voluntary and involuntary attention, with the voluntary attention being “endogenously selective”; while involuntary attention being “exogenously reflexive.” Currently, some psychologists propose that the paradigm of voluntary and involuntary attention should be revised to include a third kind of sustained attention (Bruya and Tang; Yuan, 2020a; Tang et al., 2022).

## Three kinds of attentions

In the traditional voluntary/involuntary attention, voluntary attention is regarded as effortful goal-directed, in contrary involuntary attention is drawn to outside stimulation effortlessly. However, it is interesting to note that recent scientists have suggested a third kind of attention, which uses mindfulness to manipulate attention and voluntarily focus attention to the extrovert stimulations without efforts. This kind of attention training methods has been extensively accepted as an indispensable and effective method for emotion regulation and stress reduction (Yuan, 2020b). Thus, there might be a third kinds of attention, like what the paper in this topic by Bruya and Tang, who proposed a kind of “fluid attention” or post-voluntary attention. This kind of sustained attention is voluntary, but effortless, which was drawn to the outside stimulations, like involuntary attention. This kind of mindfulness attention is characterized by dispassionate, non-evaluative and sustained moment-to-moment awareness of perceptible mental states, including continuous, immediate attention to physical sensations and affective states (Tang et al., 2015; Gu et al., 2020, 2022). Thus, there might be three kinds of attention, voluntary attention is controlled by will *via* top-down process, involuntary attention is motivated *via* emotional responses by extra stimulations, and mindfulness attention is due to will power to extrovert simulations.

## Features of mindfulness attention

One key feature about mindfulness attention is that: mindful attention is voluntary and effortful, and always involved in effortful marshaling and always involves para-sympathetic arousal (Tang et al., 2016, 2022). Just like what we previously suggested, the involuntary attention depends on emotional arousal induced sympathetic system, while the voluntary attention is related to parasympathetic system with slow heartbeat and deep breath (Gu et al., 2018). The emotional arousal induced automatic attention is mediated by LC-NE (locus coeruleus-norepinephrine), as well as the sympathetic system, which can induce automatic attention together with enhanced breath and heart rate (Gu et al., 2019a). One prominent and consistent effect of the LC-NE activation is the high motivational intensity and narrow attention, to induce conscious anchorage of involuntary attention to perform the task correctly (Gu et al., 2019b). In contrast to LC-NE system with emotional arousal, the voluntary attention is more focused on emotional valence, which can activate the parasympathetic nervous system to induce deep breath and slow heartbeat (Gu et al., 2022). On the other hand, the mindfulness attention automatically points to the environment with parasympathetic nervous (Table 1).

**Table 1:** Differences among three kinds of attention.

	**Sympathetic system**	**Parasympathetic** **system**	**Emotional** **arousal**	**Emotional** **valence**	**Voluntary** **efforts**
Voluntary attention	–	+	–	+	+
Involuntary attention	+	–	+	–	–
Mindfulness	–	+	–	–	+

## Emotion and attention

Emotion is an indispensable psychological process for attention, and attention is closely related to emotional arousal and valence (Gu et al., 2019a). Involuntary attention depends on emotion arousal, while voluntary attention depends on emotional valence (Table 1). For example, experiments have found that these ADHD children do not have trouble paying involuntary attention to something that are emotionally arousal, but they do have a difficult time to pay voluntary attention to boring, repetitive, and uninteresting tasks (Mendoza-Medialdea and Ruiz-Padial, 2021). Involuntary attention can be automatically oriented to salient stimuli in the environment, The salient stimuli might have three inborn features: threat, disgust and rewarding things, which are called three primary color model of emotions (Liang et al., 2021). To survive, animals need to find something to eat and avoid being eaten by predators (Gu et al., 2018), thus fear and joy are two major prototypical emotions, which can attract involuntary and voluntary attention, respectively (Gu et al., 2019a). The third kind of emotion disgust divert rather than attracts attention (Li et al., 2008; Zhang et al., 2017). Indeed, attention shift or diversion from a disgust object/person has been extensively accepted as an indispensable and effective method for emotion regulation and stress reduction (Yuan, 2020a,b). Thus, distraction from disgust is both a kind of rigorous voluntary attention and a kind of sustained involuntary attention, whose purpose is a kind of voluntary or involuntary diversion from a disgusting object.

## Brain loci of mindfulness attention

In addition to the automatic nervous system, many recent studies have begun to uncover the brain loci that are involved in the mindfulness attention. However, the results are inconsistent and elusive, for example, many fMRI studies have suggested that amygdala are involved in emotional arousal. On the contrary, we suggested that neuromodulators which have a wide project in the brain might be the major neural substrates for attention, for example, the LC-NE system has also been suggested to be involved in negative emotional arousal, and has been suggested to be major reason for ADHD. In addition to the fMRI, EEG studies, TMS/tDCS have also recently been used to reveal the neural mechanisms for attention, especially underlying mindfulness (Ahveninen et al., 2002; Tang et al., 2022).

In all, attention is evoked by the stimulations, which might have two features: the safety value and hedonic value. The safety feature means if the stimulation happens in an expected way, while the hedonic feature depends on if the stimulation fits into our physiological needs (Zheng et al., 2016; Wang and Tang, 2019). These two features might be related to voluntary attention (hedonic value) and involuntary attention (safety); or expected joyful stimulation can induce voluntary attention while unexpected joyful emotion can induce involuntary attention or the sustained attention (Gu et al., 2018). Disgustful emotion requires more voluntary attention, and requires more cognitive performance and more mindfulness training, and might induce the third kind of attention (van Hooff et al., 2013). Many recent studies suggested that disgustful emotion might be the major reason for most psychological disorders, thus potential applications of attention training with physical fitness, pro-social behavior and brain plasticity, as well as emotion regulation or stress reduction with mindfulness training might be a new paradigm of attention study and for the treatment of mental disorders. This Research Topic has got 17 paper through peer-reviewed process.

In the experimental paper titled “*Respiratory sinus arrhythmia during biofeedback is linked to persistent improvements in attention, short-term memory, and positive self-referential episodic memory*,” the authors Bögge et al. compared the most important biomarker heart rate variability (HRV) during voluntary attention and involuntary autonomic attentions. HRV might be decreased by parasympathetic activation, and increased by sympathetic activations. This studies provides some novel ways for measurement of the effects in mindfulness training.

In another experimental paper, Veilahti et al. introduced another study titled “*Neurofeedback learning is skill acquisition but does not guarantee treatment benefit: continuous-time analysis of learning-curves from a clinical trial for ADHD*.” This study introduced a new way of mindfulness training for ADHD, which might work as an alternative to medication. The authors suggested that mindfulness training monitored with biofeedback might be a good attention training for the ADHD patients.

In the brief research paper “*Individual differences in cognitive functioning predict compliance with restoration skills training (ReST) but not with a brief conventional mindfulness course*,” Lymeus introduced a new kind of mindfulness training, which is called restoration skills training (ReST). ReST can help regulate involuntary attention during exercise, and might offer a good way for attentional training with little attentional demands like conventional mindfulness training.

In the review paper, “*Fluid attention in education: conceptual and neurobiological framework*.” Bruya and Tang proposed a third kind of attention in addition to the voluntary and involuntary attention. The authors suggested that this third kind of attention is goal-directed like voluntary attention, and also effortlessly like involuntary attention.

In the experimental paper titled “*Measuring mind wandering during online lectures assessed with EEG*,” Conrad and Newman studied a kind of mind wandering using ERP, and found that attentional shift from the external world to internal minds might be a major reason for mind wandering.

In the review paper “*Principles of integrated cognitive training for executive attention: Application to an instrumental skill*,” Benso et al. summarized recent studies about integrated cognitive training (ICT) and suggested it might be an efficient way to train reading skills with integrated empathy, emotional arousal and avoidance of automatism.

In the experimental paper titled “*The deficit of early selective attention in adults with sluggish cognitive tempo: in comparison with those with attention- deficit/hyperactivity disorder*.” Park and Lee introduced a kind of mental disorder named sluggish cognitive tempo (SCT), which is characterized by a kind of attentional symptoms of being slow in behavior and thoughts. The study suggested that attention shifting and distractibility might be the major reason for the mental confusion and slow information processing process.

In the experimental paper titled “*Neural mechanisms of reward-by-cueing interactions: ERP evidence*.” Li X. et al. studied a kind of inhibition of return using ERP. They demonstrated that inhibition of return is inversely related to the hedonic value of the stimulation, and is also affected by voluntary attention induced by the cueing paradigm.

In the clinical study titled “*Competition enhances the effectiveness and motivation of attention rehabilitation after stroke. A randomized controlled trial*.” Navarro et al. demonstrated that voluntary motivation can help attentional rehabilitation regardless of the boring and perceived pressure and competitiveness of the projects.

In the experimental paper titled “*Event-related alpha-band power changes during self-reflection and working memory tasks in healthy individuals*,” Matsuoka et al. demonstrated that alpha-band is a biomarker for attentional problems in psychiatric disorders. They also suggested that external and internal stimulation induced alpha band might be differently affected by attentional problems.

In the experimental paper “*Attentional bias for imperfect pictures in perfectionism: An eye-movement study*,” Li J. et al. investigated the eye-tracking styles of perfectionists and found that they like to focus on imperfect tests.

In the paper titled “*Focus on the breath: Brain decoding reveals internal states of attention during meditation*,” Weng et al. found that breath and body focus meditation training can improve attentional states and also cognitive function and emotional regulation.

In the experimental paper “*Higher socioeconomic status predicts less risk of depression in adolescence: Serial mediating roles of social support and optimism*,” Zou et al. studied the psychological mechanisms of socioeconomic status induced adolescent depression, and found that high socioeconomic status can help get more social support and increased optimism.

In the experimental study, Gibson et al. submitted a paper titled “*Baseline differences in anxiety affects attention and tDCS-mediated learning*.” In the study, they found that current stimulation can effectively affect anxiety *via* both bottom up and top-down processes on attentional control and self-reported mood status.

In the experimental paper titled “*Automatic suppression reduces anxiety-related overestimation of time perception*.” Yuan et al., explored the voluntary and involuntary attentional control on the emotional regulation, and found that involuntary control is more effective in emotional control over anxiety induced emotional arousal.

In the review paper titled “Toward an individual difference perspective in mindfulness training research: Theoretical and empirical evidence.” Tang and Braver probed into empirical and theoretical evidence about the effects of mindfulness training on attention focusing, cognitive function and psychological wellbeing, and they demonstrated that pessimism personality might affect the effects of mindfulness training.

In another paper titled “*A neural marker for training focused attention meditation: closed-loop FM*θ *neurofeedback*,” Brandmeyer and Delorme showed that FMθ-meditation neurofeedback protocol over frontal electrodes can help working memory performance and attentional focus.

Altogether, this Research Topic highlights potential effects of attention-based training and its effects on attention, cognitive performance, emotional arousal, brain and physiological changes. We hope that this special topic will inspire basic and clinical research on attention-based training and develop alternative approaches with mindfulness for promoting health and preventing mental disorders.

## Author contributions

FW wrote the paper. Y-YT and RB helped with the revision. All authors contributed to the article and approved the submitted version.
